# Downregulation of TES by hypermethylation in glioblastoma reduces cell apoptosis and predicts poor clinical outcome

**DOI:** 10.1186/s40001-014-0066-4

**Published:** 2014-12-11

**Authors:** Yu Bai, Quan-Geng Zhang, Xin-Hua Wang

**Affiliations:** Department of Blood transfusion, The Central Hospital of China Aerospace Corporation, Beijing, 100049 China; Department of Immunology, Capital Medical University, Beijing, 100069 China

**Keywords:** Clinical outcome, DNA promoter methylation microarray, Glioblastoma, Testis derived transcript

## Abstract

**Background:**

Gliomas are the most common human brain tumors. Glioblastoma, also known as glioblastoma multiform (GBM), is the most aggressive, malignant, and lethal glioma. The investigation of prognostic and diagnostic molecular biomarkers in glioma patients to provide direction on clinical practice is urgent. Recent studies demonstrated that abnormal DNA methylation states play a key role in the pathogenesis of this kind of tumor. In this study, we want to identify a novel biomarker related to glioma initiation and find the role of the glioma-related gene.

**Methods:**

We performed a methylation-specific microarray on the promoter region to identify methylation gene(s) that may affect outcome of GBM patients. Normal and GBM tissues were collected from Tiantan Hospital. Genomic DNA was extracted from these tissues and analyzed with a DNA promoter methylation microarray. Testis derived transcript (TES) protein expression was analyzed by immunohistochemistry in paraffin-embedded patient tissues. Western blotting was used to detect TES protein expression in the GBM cell line U251 with or without 5-aza-dC treatment. Cell apoptosis was evaluated by flow cytometry analysis using Annexin V/PI staining.

**Results:**

We found that the *TES* promoter was hypermethylated in GBM compared to normal brain tissues under DNA promoter methylation microarray analysis. The GBM patients with *TES* hypermethylation had a short overall survival (*P* <0.05, log-rank test). Among GBM samples, reduced TES protein level was detected in 33 (89.2%) of 37 tumor tissues by immunohistochemical staining. Down regulation of TES was also correlated with worse patient outcome (*P* <0.05, log-rank test). Treatment on the GBM cell line U251 with 5-aza-dC can greatly increase TES expression, confirming the hypermethylation of TES promoter in GBM. Up-regulation of TES prompts U251 apoptosis significantly. This study demonstrated that both *TES* promoter hypermethylation and down-regulated protein expression significantly correlated with worse patient outcome. Treatment on the GBM cell line (U251) with 5-aza-dC can highly release TES expression resulting in significant apoptosis in these cells.

**Conclusions:**

Our findings suggest that the *TES* gene is a novel tumor suppressor gene and might represent a valuable prognostic marker for glioblastoma, indicating a potential target for future GBM therapy.

## Background

Gliomas are the most common central nervous system tumor in adults. Glioblastoma, also known as glioblastoma multiform (GBM), is the most aggressive, malignant, and lethal glioma. Despite radiation and temozolomide therapy, the prognosis of patients with GBM remains extremely poor, with a median survival time of only 14.5 months from diagnosis to death [1-3]. Recently, DNA methylation alterations have been widely reported in human gliomas, including global hypomethylation and promoter-associated CpG island hypermethylation [4]. To better understand the molecular mechanisms and pathological effects of genomic methylation abnormality on GBM, we performed genome-wide DNA methylation analysis on GBM (n =  42) and normal brain tissues (n  = 8). We found that the *testis derived transcript* (*TES*) gene promoter was broadly hypermethylated in GBM patients.

The *TES* gene encodes a protein (Tes) of 421 amino acids which plays a role in protein–protein interactions [5,6]. TES has an important role in cell adhesions and largely affects cell motility in a vast number of cancers [7]. According to recent studies, TES is widely considered as a putative tumor suppressor gene in malignances such as head and neck squamous cell carcinomas [8], ovarian cancer [9], primary gastric cancer [10], and prostate cancer [11]. However, TES methylation status and pathological function in GBM still remain largely unclear [12,13].

Herein, we examined the expression of TES in human GBMs and investigated the biological role of TES in the pathogenesis of this tumor. We found that TES was down-regulated in both glioblastoma cell lines and GBM tissues. TES expression level correlated inversely with the clinical outcome. Our results also indicate that down-regulation of TES protein expression is anti-apoptotic to GBM cell line.

## Methods

### Tissue samples

All samples were collected from the Department of Neurosurgery, Beijing Tiantan Hospital, between June 2009 and June 2012. Tissues were frozen in liquid nitrogen immediately after surgery; some were stored at −80°C following the extraction of RNA, whereas others were formalin-fixed and paraffin-embedded for immunohistochemistry. Informed consent from patients and ethics approval from the ethics committee was achieved. This study have been approved by the Review board of Beijing Tiantan Hospital, Capital Medical University.

### DNA methylation profiling

Methylation profiling was performed using the Illumina Infinium Methylation27 Bead Array. The BeadChip contains 27,578 highly informative CpG sites covering more than 14,000 human RefSeq genes. This allows searches to interrogate all these sites per sample at a single nucleotide resolution. Bisulfite modification of DNA, chip processing, and data analysis were carried out by following the manufacturer’s manual. DNA was extracted from peripheral-blood buffy coats using the QIAmp DNA mini kit according to the manufacturer’s protocol (Qiagen, Valencia, CA, USA); the quality of DNA samples was assessed by electrophoresis in a 1% agarose gel. DNA were then bisulfite-modified using the EZ DNA methylation kit (Zymo Research) and hybridized according to the manufacturer’s instructions.

### Immunohistochemistry

For immunohistochemistry, we used a standard avidin-biotin-peroxidase complex method. Briefly, 4-μm thick tissue sections were dewaxed and the endogenous peroxidase activity was blocked with 3% hydrogen peroxide in deionized water. For antigen retrieval, sections were immersed in an antigen repair liquid kit and then rinsed in phosphate-buffered saline (PBS; pH 7.2). The sections were incubated at 37°C for 1 h with anti-TES monoclonal antibody. After rinsing in PBS, sections were incubated with a goat anti-mouse secondary antibody for 30 min, and washed in PBS. A DAB coloration kit (ZSGB-BIO ORIGENE, ZLI-9018) was then used; PBS was used as a negative control. The percentage of positive tumor cells was counted under a bright-field Olympus microscope. Sections with fewer than 25% labeled cells indicated low expression of TES. Sections with labeling of ≥25% indicated high expression of TES.

### Cell lines and treatment with 5-Aza-2′-deoxycytidine (5-aza-dC) treatment

Glioblastoma cell lines (U251) were routinely cultured in Dulbecco’s modified eagle medium (Hyclone, Thermo Scientific, USA). The medium was supplemented with 1% L-glutamine (Hyclone), 10% fetal bovine serum (Gibco, Invitrogen Corporation, Carlsbad, CA, USA), 1% penicillin streptomycin (Hyclone), and grown at 37°C in a humidified atmosphere of 5% CO_2_/95% air. 5-aza-dC (Sigma Aldrich Corporation, St Louis, MO, USA) was dissolved in PBS (Hyclone) and filtered with a 0.22 μm filter membrane.

### Western blotting

U251 cells were lysed in 1% NP40 buffer (150 mM, 1% NP40, 50 Mm Tris, pH 8.0) following treatment with 5-aza-dC; 30 μg of protein samples were loaded onto 10% SDS-PAGE gels and then transferred to polyvinylidene fluoride membranes (Millipore). The membranes were then blocked with skimmed milk in Tris-buffered saline Tween-20 for 2 h. The following primary antibodies were used: anti-TES (Abcam, Cambridge, MA, USA) and anti-b-tubulin (Sigma-Aldrich), followed by reaction with a goat anti-mouse second antibody.

### Flow cytometry analysis of cell apoptosis

Cell apoptosis was analyzed by an Annexin V-FITC⁄propidium iodide (PI) kit according to the manufacturer’s instructions. Briefly, harvested cells were resuspended in 100 μL Annexin V-FITC binding buffer and adjusted to about 1 × 10^6^ ⁄mL, then 5 μL Annexin V-FITC and 5 μL PI (20 mg ⁄mL) were added and incubated for 15 min at room temperature in the dark. Flow cytometry was conducted on a fluorescence-activated cell sorter (FACSCalibur™ BD Biosciences, Hercules, CA, USA). Each experiment was performed in triplicate.

### Statistical analysis

Cox analysis was performed using Matlab2009. Survival curves were calculated according to the Kaplan-Meier method and differences between curves were assessed using the log-rank test. The difference between high and low TES-expressing tissues was assessed using the χ^2^ test. Significant differences among groups were determined using the Student’s *t*-test. *P* <0.05 was considered to be significant. All statistical analyses were carried out using the software GraphPad Prism (GraphPad Software, La Jolla, CA, USA) and SPSS version 16.0 (SPSS, Chicago, IL, USA).

## Results and discussion

### Identification of promoter hypermethylation of *TES* gene in GBM

**Figure 1:**
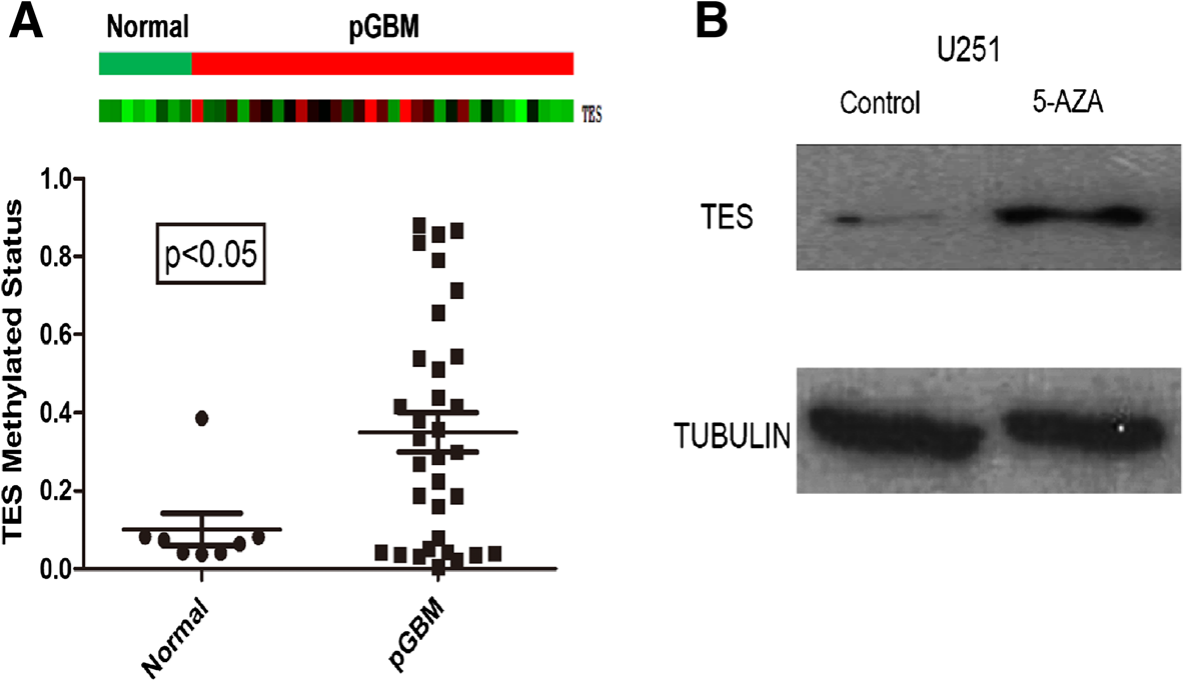
***TES*** **promoter methylation status in glioblastoma (GBM, n = 42) and normal (n = 8) samples analyzed by DNA promoter methylation microarray. (A)** Comparison of *TES* promoter methylation status in GBM and normal samples by microarray analysis (up) and independent *t*-test analysis (down). **(B)** Western blot of TES in U251 cell line before and after 5-aza-2-deoxycytidine treatment. Anti-tublin was used as a protein loading control.

We performed genome-wide DNA methylation analysis on 42 GBM and 8 normal brain tissues. To identify genes that had different methylation status between pGBM and normal tissues, all 50 methylation data were subjected to Significant Analysis of Microarray and 42 GBM were analyzed by Survival Analysis (Cox analysis) Metlab2009. Overall, 21 differential genes displayed correlation between promoter methylation and the overall survival in glioblastomas: *TUBA2*, *CKMT2*, *AMT*, *HOXC11*, *TRIM58*, *LCE1B*, *KLF14*, *SERPINB12*, *CYP2A7*, *TREML2*, *UNQ467*, *SPRR3*, *TES*, *RGN*, *DEFA1*, *AJAP1*, *PDE4C*, *SPRR2D*, *WNT7B*, *UBQLN3*, and *LCE1F*. Independent *t*-test and Kaplan-Meier method were performed on these genes to identify the differentiation and prognostic value in a univariate manner. Finally, eight genes were identified: *WNT7B*, *CKMT2*, *AMT*, *UNQ467*, *DEFA1*, *TUBA2*, *TES*, and *UBQLN3*. We selected the *TES* gene as the best candidate gene for further study (Figure 1A). To verify the hypermethylation of *TES* in GBM, treatment with demethylation reagent 5-aza-dC on the GBM cell line U251 (low TES expression) can greatly increase TES expression (Figure 1B). This result confirms the microarray data that *TES* is hypermethylated in GBM.

### Down-regulation of TES protein expression is in GBM tissues

**Figure 2:**
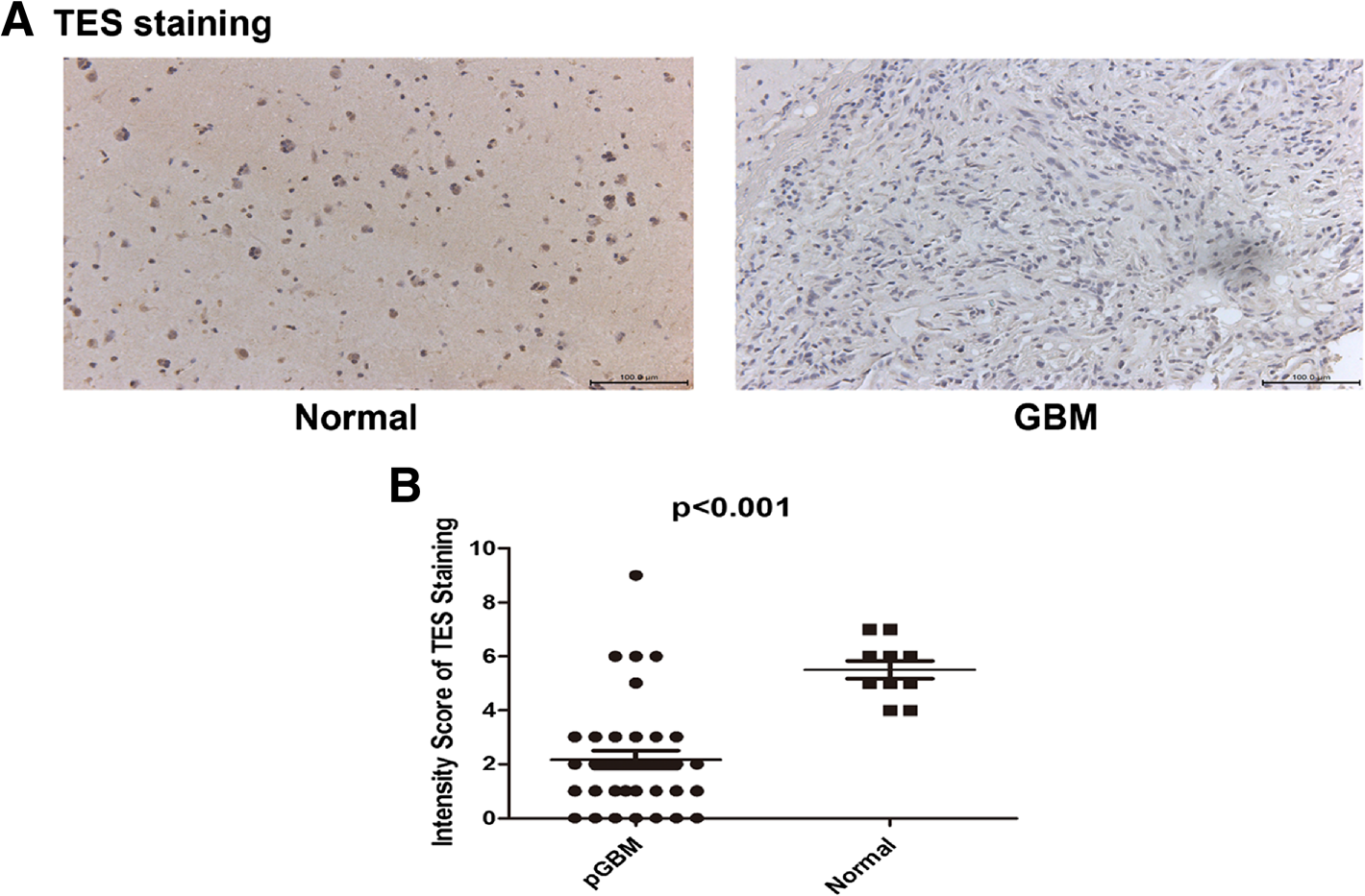
**TES expression in GBM. (A)** Immunohistochemical staining of TES protein expression in normal tissue (left) and a GBM specimen (right) (magnification × 400). **(B)** Collective summary of TES staining intensity from all samples.

TES protein expression in GBM was surveyed by immunohistochemistry using anti-TES monoclonal antibody on 37 independent GBM and 10 non-tumor samples. A reduced TES protein level was found in 33 (89.2%) tumor tissues and in 2 (20%) non-tumor tissues (*P* <0.001, Figure 2A). For evaluation of TES protein level, classification standards were as follows: high, >25% tumors cells were positively stained, low, no staining or <25% tumor cells showed negatively staining. Collectively, these results demonstrated a significant down regulation of TES protein expression in GBM (*P* <0.001, Figure 2B).

### TES enhances apoptosis in glioblastoma cells

**Figure 3:**
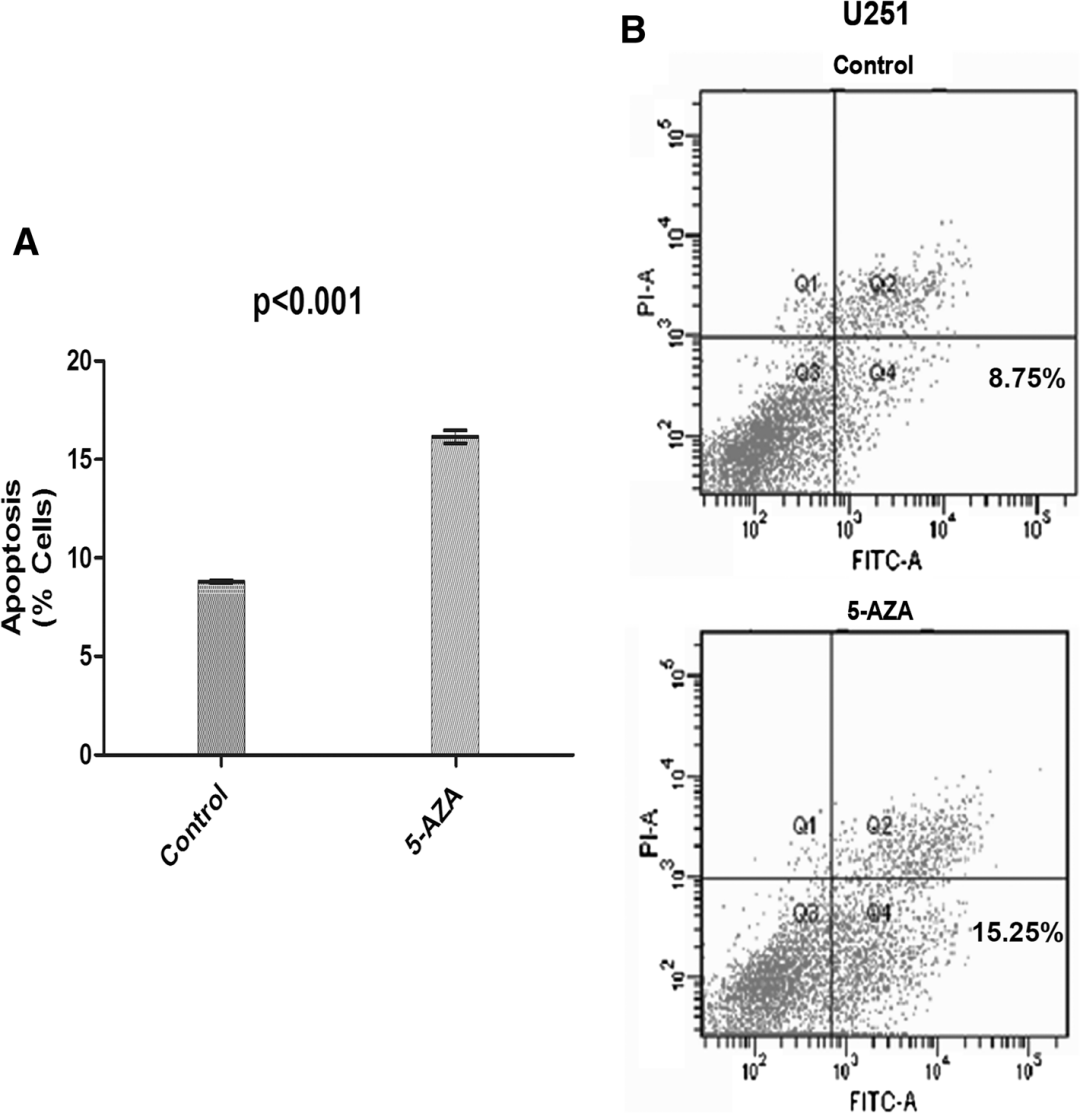
**Cell apoptosis induced by treatment of 5-aza-2-deoxycytidine.** The percentage of apoptotic cells was calculated by flow cytometry using the Annexin-V/PI double-staining assay. Control vs. 5AZA (*P <0.05). **(A)** (Control) Apoptosis of U251 cells in the group without 5-aza-dC treatment (8.75); **(B)** (5AZA) Apoptosis of U251 cells in the 5 mL 5-aza-2-dC group (15.25).

To evaluate pathogenic effect of low expression of TES in GBM, apoptotic effect was assessed on 5 μm 5-aza-dC treated and untreated U251 cells. The percentage of apoptotic cells was calculated by flow cytometry using the Annexin-V/PI double-staining assay (Figure 3A). In the untreated control group (no 5-aza-dC was given), the total rate of apoptosis was 8.75 (Figure 3B). However, in U251 cells treated with 5-aza-dC, many more apoptotic cells were detected: the rate raised to 12.25, suggesting that TES promotes apoptosis of glioblastoma cells.

### TES protein expression is highly correlated with worse outcome of GBM patients

**Figure 4:**
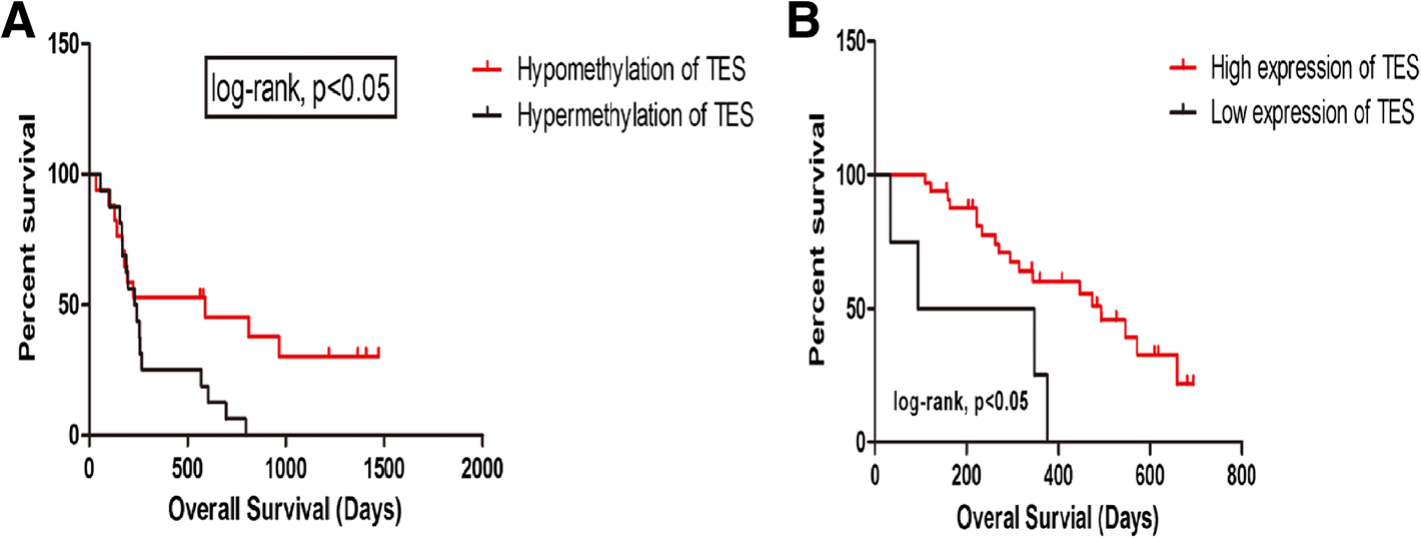
**Survial analysis according to TES expression and methylation status.** Kaplan-Meier survival estimates of overall survival according to the TES methylation status in 42 GBM patients **(A)** and according to the TES expression level in GBM patients **(B)**.

After TES promoter hypermethylation was identified in some GBM patients, we further estimated the association of this abnormality with GBM patients’ survival using Kaplan-Meier plot, as shown in Figure 4A. Results showed that the overall survival (OS) of patients with TES hypermethylation is significantly shorter than those with hypomethylation (median survival, 236.5 vs. 565 days *P* <0.05, log-rank test). Patient outcome was also evaluated independently from TES protein expression level from results of immunohistochemistry analysis. Patients with high TES expression had a median OS of 221 days, while those with low expression had an OS of 345 days (median survival, 221 vs. 345 days *P* <0.05; Figure 4B). These results suggest that the down-regulation of TES predicts worse outcome of GBM patients and that promoter hypermethylation should be the major reason for TES down-regulation, although mutation, loss of heterozygosity, etc. cannot be excluded.

The elucidation biomarkers of glioblastoma, the most common and lethal of brain tumors, has been reported since the 1990’s. Somatic mutations have been demonstrated for *TP53* on 17p [14-16], *PTEN* on 10q [17-19], *RB1* on 13q [20,21], and homozygous deletions of the *CDKN2A/p16/p14* locus on *9p* have been documented [16,22]. Other putative tumor-suppressor genes alternated at all other loci remain elusive. Methylation of promoter regions inducing silencing of gene expression, has been implicated as an alternative mechanism for tumor-suppressor gene inactivation. In glioblastomas, frequent promoter hypermethylation has been reported for *PRDX1* [23,24], *WNK2* [24], *SGNE1* [25], and *ALDH1A3* [26]. Blough et al. demonstrated that 1p Loss silence NHE-1 in oligodendroglioma [27]. Some studies showed the methylation of promoter regions with other alterations. Chou et al. identified hypermethylation of *RB1* with IDH1 and IDH2 mutation in gliomas [28].

The *TES* gene was previously identified as located in the fragile chromosomal region FRA7G at 7q31.2 [29]. Loss of TES expression has been frequently found in various cancers [5,29]. Non-synonymous mutation homozygous deletions have not been observed, consistent with CpG promoter hypermethylation being a mechanism of *TES* gene inactivation [5]. Previous studies have demonstrated that TES inhibited the growth of breast and uterine cells and promoted ovarian cancer cell apoptosis through caspase-dependent and caspase-independent processes [9,30]. Although *TES* could be a candidate tumor suppressor gene in glioma cells, the mechanism of this role is still unknown.

TES, a highly conserved protein, consists of three C-terminal LIM domains and a PET (prickle, espinas, and testin) domain of unknown function. Group 3 LIM domain proteins, including the TES domain, are a component of the focal adhesion complex and localize to cell-matrix adhesions and cell-cell contacts [31]. Over-expression of TES increases cell spreading and decreased cell motility [32]. Adenoviral transduction of the *TES* gene into breast and uterine cancer cell lines promotes apoptosis and tumor reduction [30]. Further functional analysis demonstrated that *TES* acted as a tumor suppressor gene *in vivo*, as *TES* knockout mice showed an increased susceptibility to induced gastric cancer [6]. Our results in this study showed that *TES* is widely down-regulated in GBM. Elevation of TES expression can increase apoptosis of GBM cells.

TES can be silenced either by loss of heterozygosity or by promoter hypermethylation in several tumor types, including head and neck squamous cell carcinoma, ovarian cancer, primary gastric cancer, prostate cancer, breast cancer, among others [8-12].

Our data suggests promoter hypermethylation is the major cause of down-regulation or silencing of *TES* in GBM, even if other mechanisms cannot be excluded at this point.

## Conclusions

Using whole-genome DNA methylation microarray we found that the *TES* gene is a novel tumor suppressor gene and might represent a valuable prognostic marker for glioblastoma, indicating a potential future target for GBM therapy.

## Abbreviations

GBM: Glioblastoma multiform
5-aza-dC: 5-Aza-2′-deoxycytidine
OS: Overall survival
PBS: Phosphate-buffered saline
PI: Propidium iodide
TES: Testis derived transcript
